# Supplementary material for: Establishment of a Gene Signature to Predict Prognosis for Patients with Lung Adenocarcinoma
Source: Int J Mol Sci. 2020 Nov 11;21(22):8479. doi: 10.3390/ijms21228479 (PMC7697394; doi:10.3390/ijms21228479)
Supplement: Supplementary file 1 [file ijms-21-08479-s001.zip › Supplementary materials/Supplementary Files_Figure.docx]

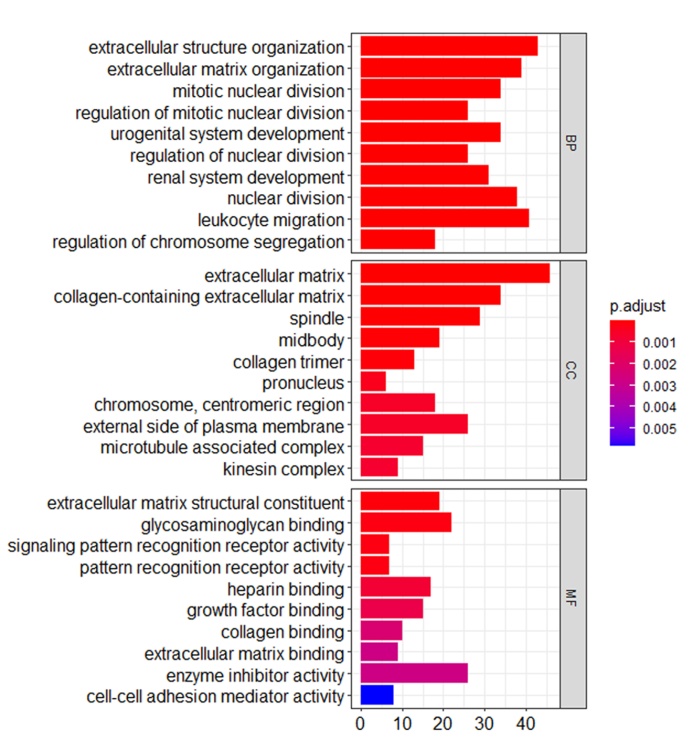

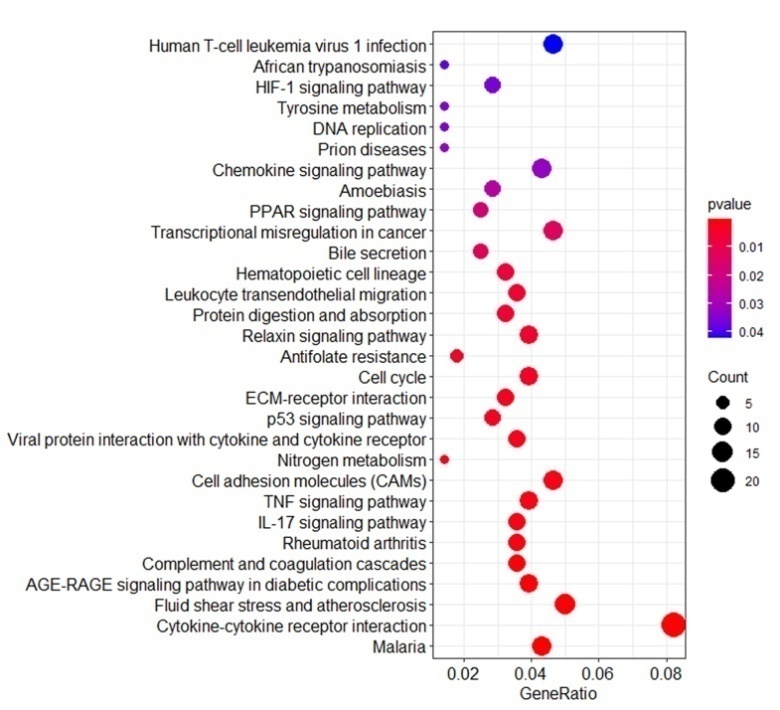


B

A

**Figure S1.** GO enrichment analysis (A) and KEGG pathway analysis (B) were conducted based on the 573 shared DEGs.


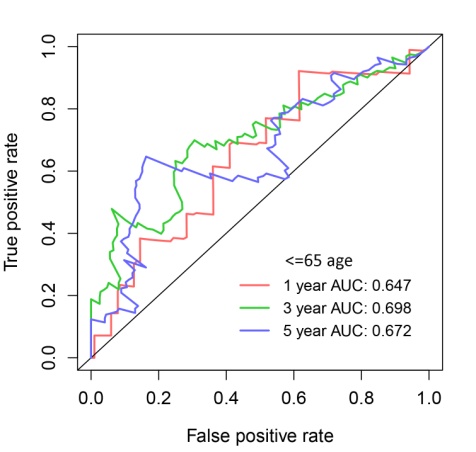

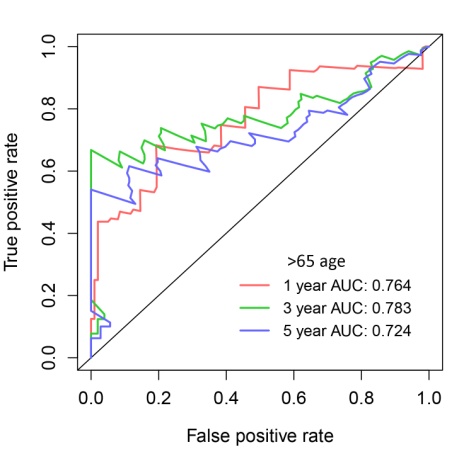

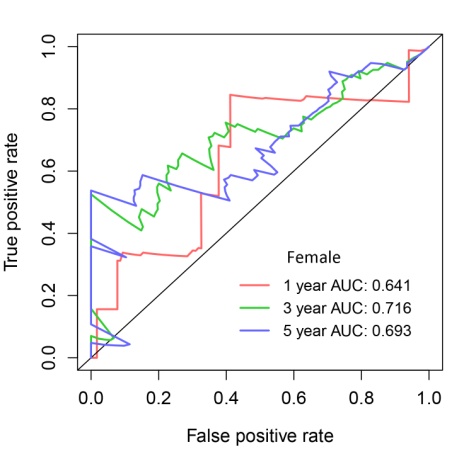


D

F

E


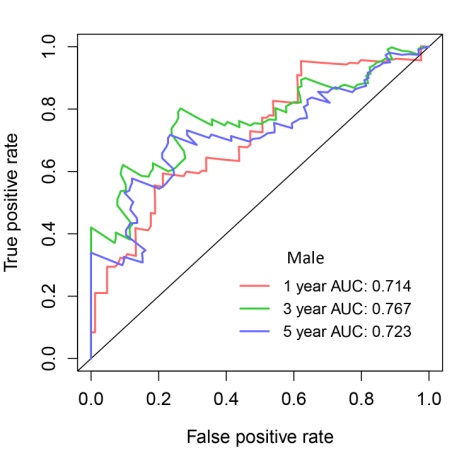

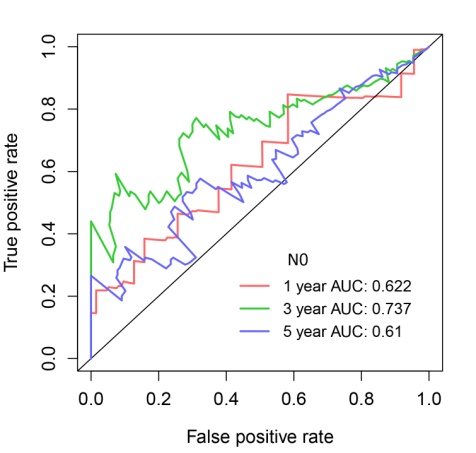

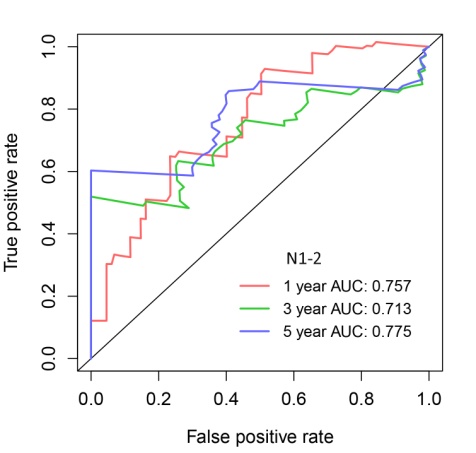


I

H

G


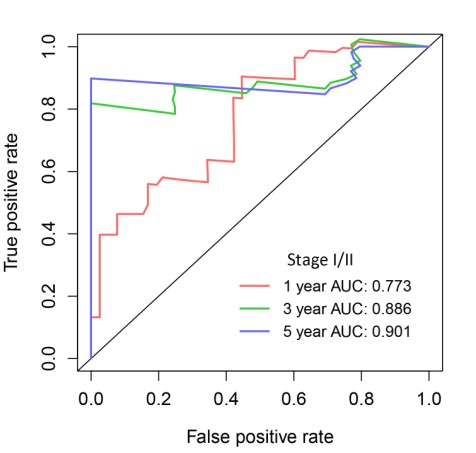

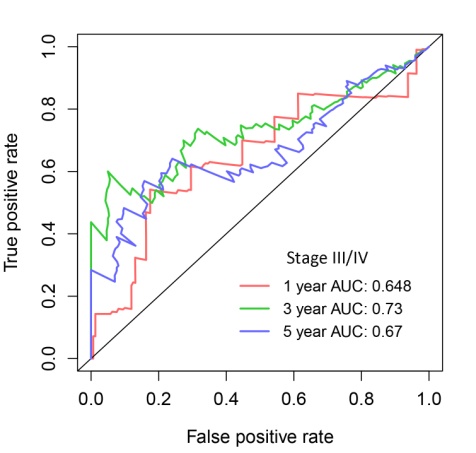

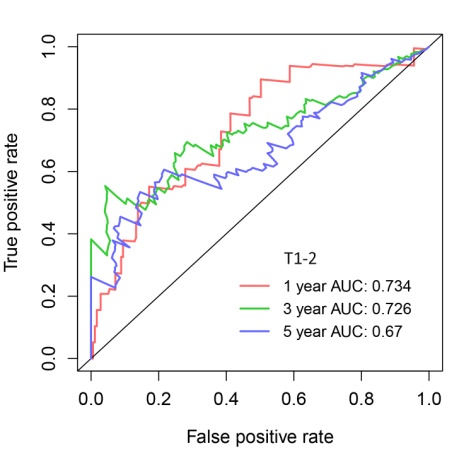


J


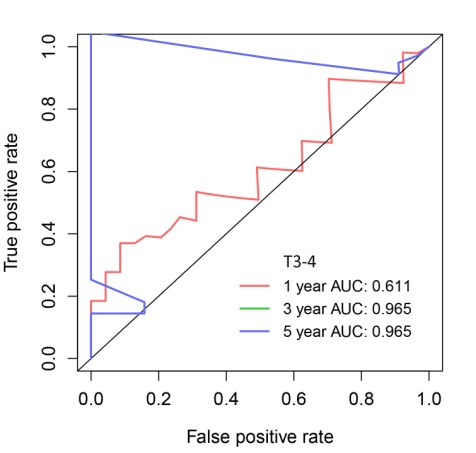


C

B

A

**Figure S2.** The prognostic value of risk score signature evaluated by ROC curves at 1, 3 and 5 years in age:<=65 (A) and >65 (B), gender: female (C) and male (D), clinical stage I/II (E) and III/IV (F), lymph node metastasis:N0 (G) and N1-3 (H), and primary tumor:T1-2 (I) and T3-4 (J) subgroups.

**tumor**

**normal**

**tumor**

**normal**

A


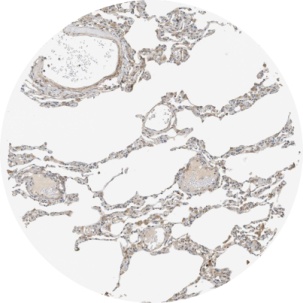

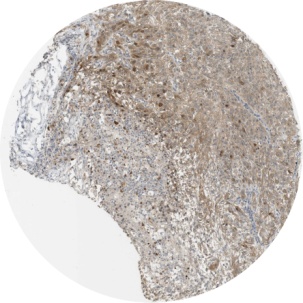

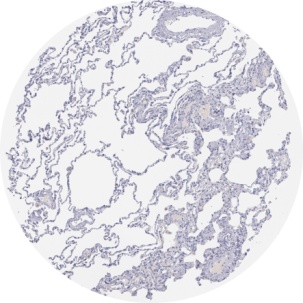

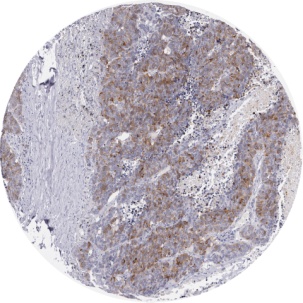


HMMR

（CAB002433）

TTK

（CAB013229）


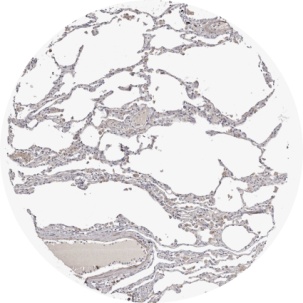

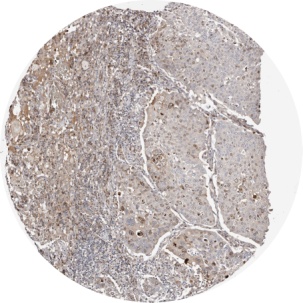

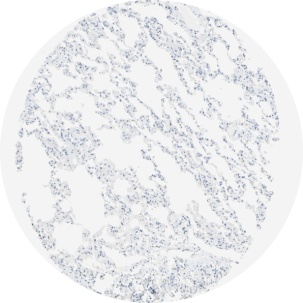

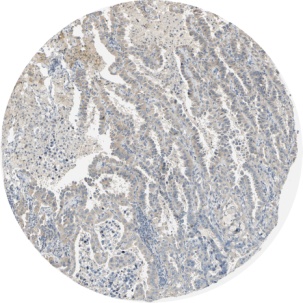


CCNB2

（CAB009575）

CDCA8

（HPA028258）


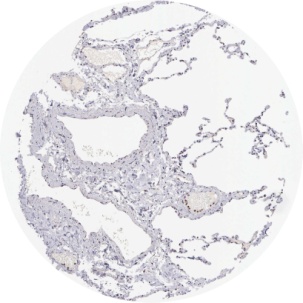

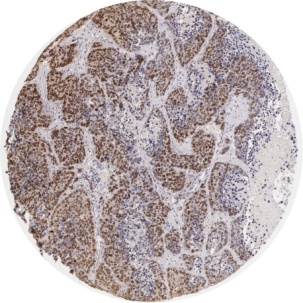

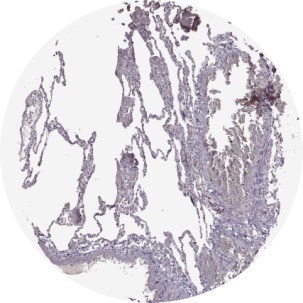

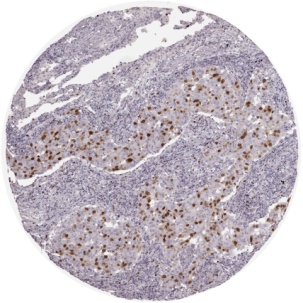


CCNA2

（CAB000114）

MKI67

（HPA000451）

B


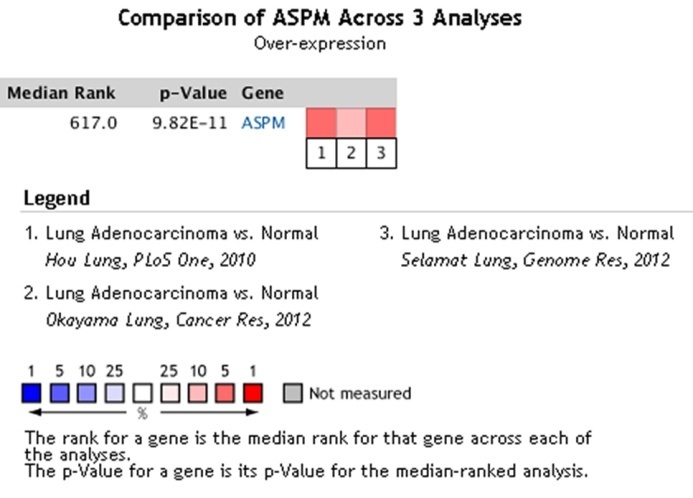

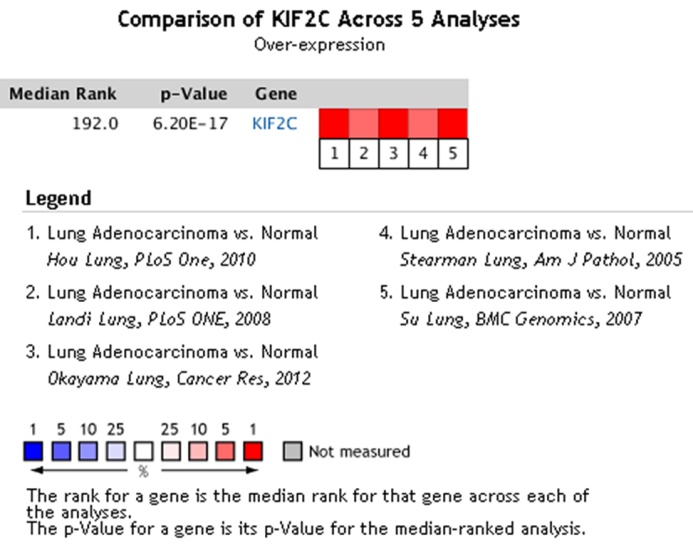


**Figure S3.** Validation of eight core genes in risk score model. A. IHC for corresponding genes expression in LUAD and normal lung tissues retrieved from HPA database. And the protein levels were obviously up-regulated in LUAD tissues for the six genes. B. Meta-analysis for ASPM and KIF2C expression based on Oncomine platform, which showed that both mRNA levels were increased in LUAD versus normal lung tissues. And red and blue color represent gene over-expression and under-expression, respectively.


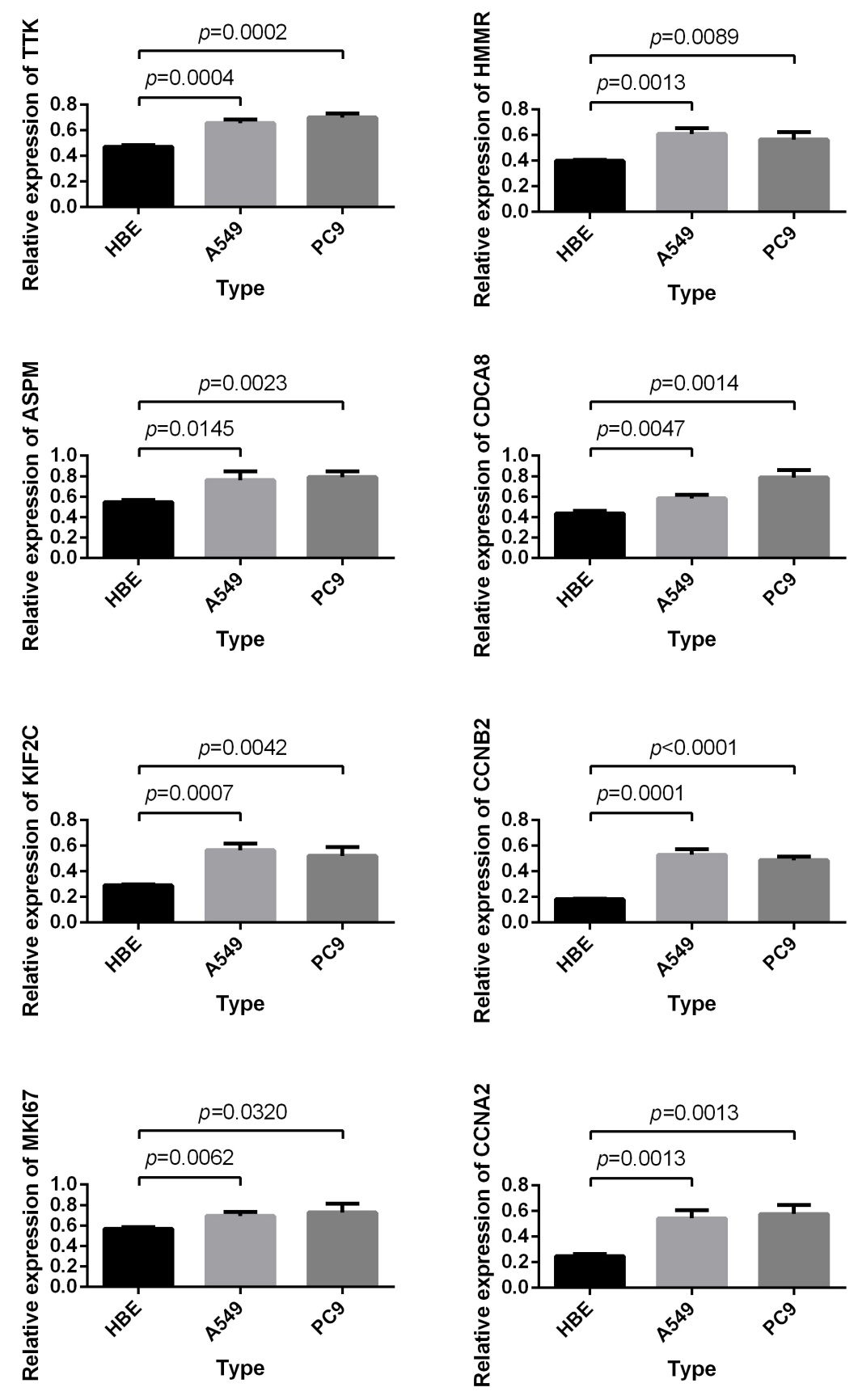


**Figure S4.** Relative quantification of protein expression in HBE, A549, and PC-9 cell lines. The protein levels of TTK, HMMR, ASPM, CDCA8, KIF2C, CCNB2, MKI67, and CCNA2 were significantly increased in A549 and PC-9 versus HBE (*p*<0.05). The measurements of protein expression were performed in triplicate.
